# Acute trauma induced disc displacement without reduction and its sequelae

**DOI:** 10.1038/srep32684

**Published:** 2016-09-01

**Authors:** DongMei He, XiuJuan Yang, FeiYu Wang, Chi Yang, MinJun Dong

**Affiliations:** 1 Department of Oral Surgery, Ninth People’s Hospital, College of Stomatology, Shanghai Jiao Tong University School of Medicine, Key Laboratory of Stomatology, Shanghai, 200011, China; 2 Department of Radiology, Ninth People’s Hospital, Shanghai Jiao Tong University School of Medicine, Shanghai, 200011, China

## Abstract

Acute traumatic temporomandibular joint disc displacement (ATDD) and its sequelae are not familiar for most surgeons. This study is to discuss its sequelae in cases without disc reduction after failed conservative treatment. From 2010 to 2015, 26 patients with 34 joints were included in the study. All patients had at least 3 months conservative treatment. Their maximal incisor opening (MIO) was measured during follow-ups and MRI examination was used to check the condylar bone degeneration. The mean follow-up for conservative treatment after admission was 8.69 months, the patients reached an average of 25.7 mm MIO. MRI showed condylar bone intact in 8 joints (23.5%), condylar surface bone destruction (Wilks IV, V stages) in 14 joints (41.2%), and severe bone resorption in 12 joints (35.3%). 15 patients with 23 joints were asked for surgical treatment after a mean conservative treatment of 5.4 months (3–12 months) to improve mouth opening and relieve chronic pain. 12 joints had total joint replacement (TJR). 11 joints had disc repositioning. Their mean MIO before operation was 19.8 mm and significantly improved to 33.9 mm after operation (p = 0.0000). ATDD may cause severe osteoarthritis or ankylosis. Disc repositioning and TJR could significantly improve MIO.

Trauma to the temporomandibular joint (TMJ) can cause both bone and soft tissue injuries, like intracapsular condylar fracture (ICF) with mostly displaced TMJ disc without reduction^1^,^2^,^3^,^4^,^5^. Whereas there is another type of injury without condylar fracture, only displaced disc without reduction^4^,^6^,^7^. This is very easy to neglect because disc displacement can not be detected in computer tomography (CT) or X-ray films. The development and application of magnetic resonance imaging (MRI) provided valuable information on both bone and soft tissue injuries. Our previous study^8^ showed that the characteristics of ATDD without reduction are: (1) facial trauma, especially in the chin area; (2) no TMJ pain, clicking, crispus, mouth opening limitation and mandibular movement dysfunction before injury; (3) TMJ swelling, pain and mouth opening limitation after injury; (4) CT examination showing no condylar fracture on the painful side, but a decreased or diminished space between the condyle and fossa on the coronal reconstruction; and (5) MRI examination showing an anteriorly displaced TMJ disc with a normal shape, but the posterior disc band is elongated or maybe disrupted with or without effusion in the joint space. This is different from the patients who have physiologically displaced disc according to MRI but without any clinical signs. The length and shape of those patients’ disc are not normal, usually deformed.

Disc displacement by acute trauma may develop much faster than the one by micro trauma. Merrill^2^ used TMJ arthroscopy to check 1151 patients with internal derangement and showed that 60% had a history of mandibular trauma at least one year before. Although patients had no condylar fracture, there was severe condylar surface bone destruction with an anteriorly displaced TMJ disc. Another study by Wang^9^ showed that discs became displaced in between 18% and 66% of cases when there was no condylar fracture in the 22 patients after acute mandibular injury. The mandibular condyle and articular tubercle were impaired in approximately 20% of cases. This is different from Kolk and Neff’s study that osteoarthritis happened around 9.1~11.5% in ICFs^10^. Because most of the time, the TMJ disc displaces with the condylar head, so the condylar cartilage is well protected. But in ATDD cases, without disc protection, the condylar cartilage may degenerate fast. As well known in the literature and also in clinical practice, as much as at least 25% of the disc displacement without reduction cases described in MRI are in fact physiological variants without presenting any pathology. The clinical symptoms are not consistent with the MRI findings.

Although conservative treatment could provide some 80% sufficient healing in the micro-trauma caused disc displacement^11^,^12^, there were no reports on the prognosis of disc displacement caused by acute trauma. This was dealt with so far in cases with concomitant fractures^10^. Is it developed faster and need early surgical treatment to prevent osteoarthritis or ankylosis are still unknown.

This study sought to find the sequelae of ATDD without reduction after failed conservative treatment by both clinical and MRI evaluation.

## Results

### Basic information

**Figure 1:**
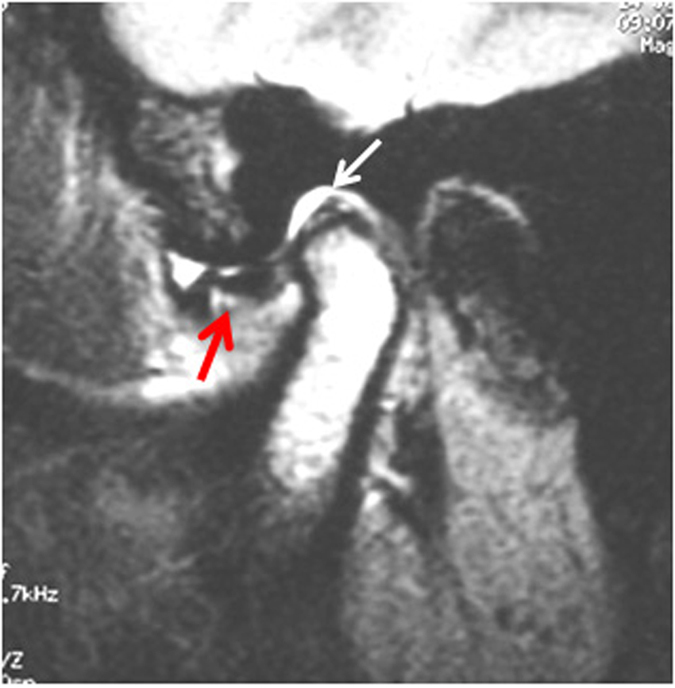
MRI showed acute phase of ATDD, disc displaced in front of the condyle (red arrow) with normal shape and length. The posterior band was disrupted and elongated with or without effusion (white arrow).

Twenty-six patients with 34 joints were included in the study (Table 1). They all had disc displacement without reduction by MRI examination (Fig. 1). Among them, there were 14 males and 12 females, with an age ranged from 4 to 49 years old (mean, 32.6 years). One patient with bilateral ATDD without Reduction also had a combined mandibular symphysis fracture which was reduced and rigid fixed immediately after injury. But his bilateral ATDD developed to osteoarthritis after 6 months conservative treatment. Four patients had one joint of ATDD, and the other joint of ICF. One of the 4 ICFs developed to ankylosis after 4 months conservative treatment and accepted costochondral graft for joint replacement. The other 3 ICFs healed with malunion of the ramus. The follow-up period was from 3 to 24 months (mean, 8.69 months).

### Treatment and follow-ups

MIO during the last follow-up was from 7 to 40 mm (mean 25.7 mm).

**Figure 2:**
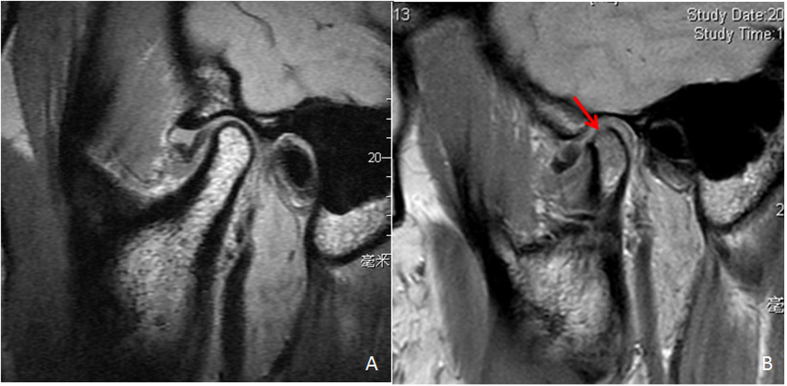
MRI showed condylar bone degeneration after ATDD. (**A**) disc displaced anteriorly 3 weeks after injury, the condylar bone was intact. (**B**) the condylar surface bone resorbed 5 months after injury (arrow).

**Figure 3:**
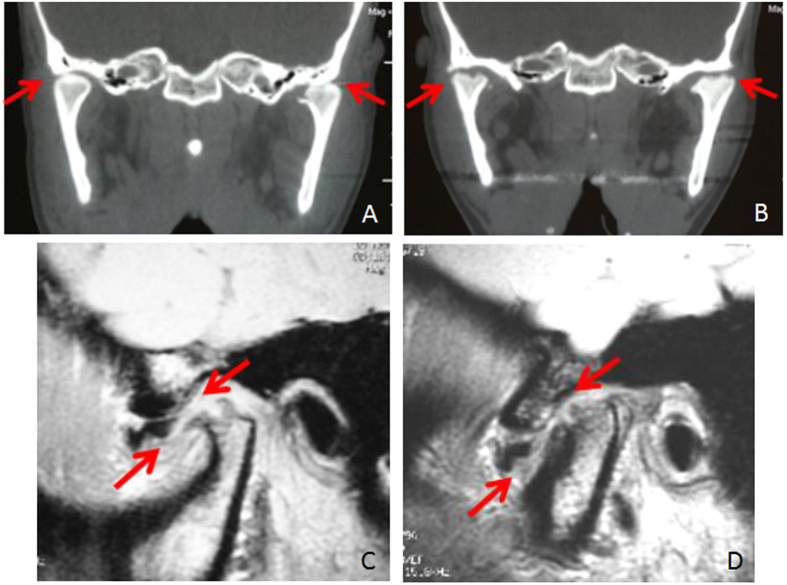
Disc displacement and condylar bone destruction in the end stage. (**A**) Decreased joint space post injury by coronal CT reconstruction (arrow). (**B**) Condylar bone resorption 3 months post injury by coronal CT reconstruction. (**C**) MRI showed right disc displacement with condylar bone destruction (arrow). (**D**) MRI showed left disc displacement with condylar bone destruction (arrow).

At the end of conservative follow up, MRI showed condylar bone intact in 6 patients with 8 joints (23.5%), condylar surface bone destruction (Wilks IV, V stages) in 13 patients with 14 joints (41.2%, Fig. 2), and severe bone resorption in 7 patients with 12 joints (35.3%, Fig. 3, Table 1).

15 patients with 23 joints were asked for surgical treatment after a mean conservative treatment of 5.4 months (3–12 months) to improve mouth opening and relieve chronic pain (Table 2). 7 patients with 12 joints had total joint replacement (TJR). 8 patients with 11 joints had disc repositioning. Their mean MIO before operation was 19.8 mm. After operation their mean MIO was significantly improved to 33.9 mm (p = 0.0000, Table 2).

## Discussion

Disc displacement can be caused by micro-trauma or acute trauma. There are many reports on temporomandibular disorders (TMD) caused by micro-trauma, but few on ATDD without reduction. Doctors usually pay more attention to whether there is a condylar fracture and overlook cases without a condylar fracture^6^,^7^. Are those intact condyles really healthy? After acute trauma, TMJ changes and the governing factors in the development of complications are rarely reported. There is also a lack of reports on the long-term follow-up results of ATDD without reduction^9^. Although conservative treatment for the disc displacement without reduction showed some good results^11^,^12^, the prognosis is quiet different in the acute trauma. This study showed that the condylar bone degenerated fast after at least 3 months conservative treatment, 35.3% of the condyle had severe bone resorption and adhesion in the joint which caused chronic pain and mouth opening limitation. TJR was selected for treatment. This is different from Kolk and Neff’s study that osteoarthritis happened around 9.1~11.5% in ICFs^10^. Because most of the time, the TMJ disc displaces with the condylar head, so the condylar cartilage is well protected. But in this study, without disc protection, the condylar cartilage degenerated fast. As well known in the literature and also in clinical practice, as much as at least 25% of the disc displacement without reduction cases described in MRI are in fact physiological variants without presenting any pathology. The clinical symptoms are not consistent with the MRI findings. In this study, we only included patients with failed conservative treatment and found the high incidence of bone destruction with or without clinical symptoms.

Posttraumatic osteoarthritis (PTOA) is the most severe and fast developing type of osteoarthritis due to direct trauma to the mandible (mostly the chin), which causes damage to the articular cartilage; a tear of the TMJ retrodiscal lamina and an anterior acute disc displacement. Because of the backward force to the condyle, the disc is severely extruded, which may result in a retrodisc or lateral disc ligament rupture and the squeezing of the disc body to the anterior part of the condyle, causing acute disc displacement. The cartilage is damaged with subchondral bone microfracture, causing minor sinking. Due to the lack of protection of the disc body (role of stress distribution), the occurrence and development of condylar osteoarthritis are rapid. Some patients developed end-stage disease very fast in this study. TJR was performed for those patients to relieve chronic pain and improve mouth opening.

The clinical and experimental results showed that disc displacement could cause an increased load on the condyle and promote it to resorb and degenerate to osteoarthritis or even ankylosis^13^,^14^,^15^,^16^,^17^,^18^,^19^,^20^,^21^. Our previous clinical observation showed that disc repositioning could stimulate condyle growth in growing patients^16^. However, the results of disc repositioning on the early stage after trauma are unknown. In this study, 8 patients with 11 joints accepted disc repositioning after at least 3 months failed conservative treatment. Their mouth opening was significantly improved compared before operation. No severe condylar bone resorption was happened after disc repositioning in this study. Abramowicz also reported in 2010 that TMJ disc repositioning is an effective and successful surgical treatment for TMJ internal derangement, which can be maintained for 20 years^18^. Disc repositioning according to an animal model presented by Li and He *et al*.^17^ can prevent severe osteoarthritis and ankylosis.

In conclusion, ATDD may cause severe osteoarthritis or ankylosis. Disc repositioning and TJR could significantly improve MIO. It may be supposed that patients not responding adequately to conservative treatment are more susceptible to develop OA or even ankylosis. Further studies are required to analyse the underlying factors responsible for adverse outcomes after ATDD.

## Patients and methods

### Clinical data

This was a cohort retrospective study. All methods were carried out in accordance with relevant guidelines and regulations. It was approved by the local ethics board of the hospital (Scientific Research Projects Approval Determination of Independent Ethics Committee of Shanghai Ninth People’s Hospital affiliated to Shanghai JiaoTong University, School of Medicine, 2014–46). The informed consent was obtained from all subjects. Patients who had pre-auricular pain and a mouth opening limitation after facial trauma from January 2010 to December 2015 were included in the study. The criteria for the diagnosis of ATDD were: (1) history of facial trauma; (2) no TMJ symptoms, such as pain, clicking, crepitus, mouth opening limitation, and abnormal mandibular movement before trauma but persistent pre-auricular pain after trauma; (3) no condylar fracture on the painful side by CT examination; (4) MRI examination showed an anteriorly displaced TMJ disc with a normal or nearly normal shape and length; (5) Patients were excluded if they had: (1) TMJ symptoms before trauma; (2) condylar fracture; and (3) abnormal disc shape and length in MRI.

### Treatment and evaluation

Conservative treatment including mouth opening exercise and medication to relieve pain were used. MIO and condylar bone degeneration was evaluated by MRI durging follow-ups. For the patients failed of conservative treatment more than 3 months, disc repositioning or TJR was used according to the TMJ status to improve MIO and relieve pain for the patients. MIO before and after operation was compared using Statistical Package for Social Sciences, version 13.0 (SPSS, Chicago, IL). Student’s paired *t* test was used to establish intragroup (before and after treatment) significance and non-parametric tests on k independent samples, and Kruskal-Wallis *H* tests were used to determine the significance of intergroup MIO improvement. An α level of ≤0.05 was considered significant.

## Additional Information

**How to cite this article**: He, D.M. *et al*. Acute trauma induced disc displacement without reduction and its sequelae. *Sci. Rep.* **6**, 32684; doi: 10.1038/srep32684 (2016).

## Figures and Tables

**Table 1:** Basic information and conservative treatment results.

Number of patients (Joints)	26 (34)
Gender	14 males, 12 females
Age	32.6 ± 11.4 (4~49) years old
Follow-up period	8.69 ± 6.4 (3~24) months
MIO	25.7 ± 9.6 (7~40)mm
Condylar cortical bone continuity	6 (8, 23.5%)
Condylar cortical bone incontinuity	13 (14, 41.2%)
Condylar severe bone resorption	7 (12, 35.3%)

**Table 2:** Surgical treatment results.

	Disc reposioning	Total Joint replacement
Number of patients (Joints)	8 (11)	7 (12)
Time point of surgical treatment	3–12 months (mean 5.4 months)	
MIO before operation	19.8 ± 8.2 (7~32)mm*	
MIO after operation	33.9 ± 5.9 (25~45)mm*	

^*^p = 0.000
